# Complex Correlation Measure: a novel descriptor for Poincaré plot

**DOI:** 10.1186/1475-925X-8-17

**Published:** 2009-08-13

**Authors:** Chandan K Karmakar, Ahsan H Khandoker, Jayavardhana Gubbi, Marimuthu Palaniswami

**Affiliations:** 1 Department of Electrical and Electronic Engineering, University of Melbourne, Melbourne, VIC 3010, Australia

## Abstract

**Background:**

Poincaré plot is one of the important techniques used for visually representing the heart rate variability. It is valuable due to its ability to display nonlinear aspects of the data sequence. However, the problem lies in capturing temporal information of the plot quantitatively. The standard descriptors used in quantifying the Poincaré plot (*SD*1, *SD*2) measure the gross variability of the time series data. Determination of advanced methods for capturing temporal properties pose a significant challenge. In this paper, we propose a novel descriptor "Complex Correlation Measure (*CCM*)" to quantify the temporal aspect of the Poincaré plot. In contrast to *SD*1 and *SD*2, the *CCM *incorporates point-to-point variation of the signal.

**Methods:**

First, we have derived expressions for *CCM*. Then the sensitivity of descriptors has been shown by measuring all descriptors before and after surrogation of the signal. For each case study, *lag-1 *Poincaré plots were constructed for three groups of subjects (Arrhythmia, Congestive Heart Failure (CHF) and those with Normal Sinus Rhythm (NSR)), and the new measure *CCM *was computed along with *SD*1 and *SD*2. ANOVA analysis distribution was used to define the level of significance of mean and variance of *SD*1, *SD*2 and *CCM *for different groups of subjects.

**Results:**

*CCM *is defined based on the autocorrelation at different lags of the time series, hence giving an in depth measurement of the correlation structure of the Poincaré plot. A surrogate analysis was performed, and the sensitivity of the proposed descriptor was found to be higher as compared to the standard descriptors. Two case studies were conducted for recognizing arrhythmia and congestive heart failure (CHF) subjects from those with NSR, using the Physionet database and demonstrated the usefulness of the proposed descriptors in biomedical applications. *CCM *was found to be a more significant (*p *= 6.28E-18) parameter than *SD*1 and *SD*2 in discriminating arrhythmia from NSR subjects. In case of assessing CHF subjects also against NSR, *CCM *was again found to be the most significant (*p *= 9.07E-14).

**Conclusion:**

Hence, *CCM *can be used as an additional Poincaré plot descriptor to detect pathology.

## Background

**Figure 1:**
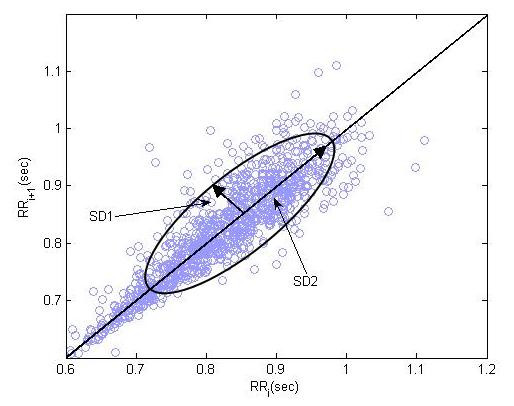
**Standard Poincaré plot**. A standard Poincaré plot (*lag-1*) of RR intervals of a healthy person (N = 2000). *SD*1 and *SD*2 represents the dispersion along minor and major axis of the fitted ellipse.

Poincaré plot is a geometrical representation of a time series in a Cartesian plane. It has been shown to reveal patterns of heart rate dynamics resulting from nonlinear processes [1,2]. A two dimensional plot constructed by plotting consecutive points is a representation of RR time-series on phase space or cartesian plane [3]. Poincaré plot is extensively used for qualitative visualization of physiological signal. It is commonly applied to asses the dynamics of heart rate variability (HRV) [1,4-7]. Tulppo *et. al. *[1] fitted an ellipse to the shape of the Poincaré plot and defined two standard descriptors of the plot *SD*1 and *SD*2 for quantification of the Poincaré plot geometry. These standard descriptor represent the minor axis and the major axis of the ellipse respectively as shown in figure 1. The description of *SD*1 and *SD*2 in terms of linear statistics, given by Brennan *et. al. *[2], shows that the standard descriptors guide the visual inspection of the distribution. In case of HRV, it reveals a useful visual pattern of the RR interval data by representing both short and long term variations of the signal [1,2]. The inherent assumption behind using consecutive RR points is that the "present-RR-interval" significantly influences the "following-RR-interval". Various authors have shown that varying lags of Poincaré plot give better understanding about the autonomic control of the heart rate that influence the short term and long term variability of the heart rate [8,9]. A system can have different short and long term correlations on different time scales. When the sampling interval is less than the short time correlation length, then these short time correlations can be predominantly seen [10]. So in the context of short or long term variability any point can influence at least few successive points. Lerma *et. al. *[11] reported that the current RR interval can influence up to approximately eight subsequent RR intervals in the context of the short term variability. In another study [12], the authors examined the theoretical demand with different lags and showed that there is a curvilinear relationship between lag Poincaré plot indices for normal subjects, which is lost in Congestive Heart Failure (CHF) patients. Therefore, measurement from a series of lagged Poincaré plots (multiple lag correlation) can potentially provide more information about the behavior of Poincaré plot than the conventional *lag-1 *plot measurements [11].

As mentioned earlier, standard descriptors *SD*1 and *SD*2 are linear statistics [2] and hence the measures do not directly quantify the nonlinear temporal variations in the time series contained in the Poincaré plot. Further, when applied to the data sets that form multiple clusters in a Poincaré plot due to complex dynamic behaviors, the *SD*1/*SD*2 statistics yields mixed results. This is because the technique relies on the existence of a single cluster or a defined pattern [13,14]. Moreover, the limitations of the *SD*1/*SD*2 analysis are important to understand when attempting to investigate the physiological mechanisms in a time series, or when analyzing data where the occurrence of nonlinear behavior may be a distinguishing feature between health and disease. Such a study will also enable further studies in defining new descriptors for Poincaré plot which is currently not being addressed by researchers in this field. The necessity for such a study arises from the fact that the visual pattern is relied upon clinical scenarios and the application of the existing standard descriptors in various studies has resulted in limited success.

Therefore, we hypothesize that any descriptor that captures temporal information and is a function of multiple lag correlation, would provide more insight into the system rather than conventional measurements of variability of Poincaré plot (*SD*1 and *SD*2), which is a function of a *lag-1 *correlation. In this study, we propose a novel descriptor for Poincaré plot that can be applied to measure the multiple lag correlation of the signal. Unlike *SD*1, *SD*2 and *SD*1/*SD*2 terms, the proposed measure incorporates the temporal information of the time series. In this paper, we aim to evaluate all three descriptors (*SD*1, *SD*2 and *CCM*) of the Poincaré plot of RR intervals, and compare their performance in differentiating arrhythmia and CHF from normal subjects.

### Standard Poincaré Plot Analysis

This section describes the standard descriptors of Poincaré plot and their limitations. In this paper, we have used RR interval time series signal to plot the Poincaré plot which is denoted by *RR*_*n*_. We assume that a finite number of RR intervals are available and a wide-stationarity of the RR interval as suggested in literature [2].

#### Standard Descriptors

A standard Poincaré plot of RR interval is shown in figure 1. Two basic descriptors of the plot are *SD*1 and *SD*2 and their mathematical derivation can be found in [2]. The line of identity is the 45° imaginary diagonal line on the Poincaré plot and the points falling on the imaginary line has the property *RR*_*n *_= *RR*_*n*+1_. *SD*1 measures the dispersion of points perpendicular to the line of identity, whereas *SD*2 measures the dispersion along the line of identity. Fundamentally, *SD*1 and *SD*2 of Poincaré plot is directly related to the basic statistical measures, standard deviation of RR interval (SDRR), and standard deviation of the successive difference of RR interval (SDSD), which is given by the relation shown in equation 1 and equation 2.

(1)

(2)

where _*γRR*_(0) and _*γRR *_(1) is the autocorrelation function for *lag-0 *and *lag-1 RR *interval and  is the mean of RR intervals. From equations 1 and 2, it is clear that the measures *SD*1 and *SD*2 are actually derived from the correlation and mean of the RR intervals time series with *lag-0 *and *lag-1*. The above equation sets are derived for unit time delay Poincaré plot. Researchers have shown interest in plots with different time delays to get a better insight in the time-series signal. Usually the time delay is multiple of the cycle length or the sampling time of the signal [15]. The dependency among the variables are controlled by the choice of time delay, and the most conventional analysis is performed with higher order linear correlation between points. In case of plotting the 2*D *phase space with *lag-m *the equations for *SD*1 and *SD*2 can be represented as:

(3)

and

(4)

where _*γRR *_(*m*) is the autocorrelation function for *lag-m *RR interval. This implies that the standard descriptors for any arbitrary *m *lag Poincaré plot is a function of autocorrelation of the signal at *lag-0 *and *lag-m*.

#### Limitations of Standard Descriptors

**Figure 2:**
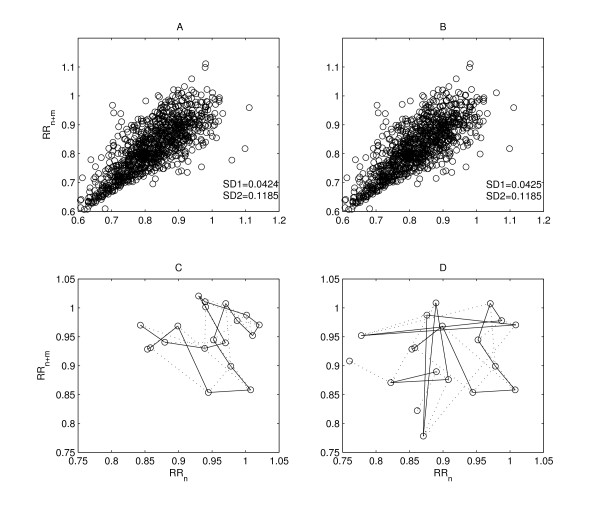
**Underlying temporal dynamics of a Poincaré plot**. Poincaré plots with similar *SD*1 and *SD*2 values with different temporal structure are shown. Top panel (A and B) shows the Poincaré plots (*lag-m *= 2) of two different RR intervals series of length N (N = 1000) with *SD*1 (0.0424 and 0.0425) and *SD*2 (0.1185 and 0.1185) values. The underlying temporal dynamics of first 20 points of the same RR intervals are shown in the bottom panel (C and D), which shows the visible difference among them.

The lack of temporal information is the primary limitation of the standard descriptors of the Poincaré plot. *SD*1 and *SD*2 represents the distribution of signal in 2D space and carries only spatial (shape) information. It should be noted that many possible RR interval series result in identical plot with exactly similar *SD*1 and *SD*2 values in spite of different temporal structure. In figure 2, two signals with similar *SD*1 and *SD*2 values are shown to be different in terms of temporal structure (bottom panels).

In a study [11], authors have shown that the measurement from multiple lag Poincaré plot provides more information than any measure from single lag Poincaré plot. Indeed, the Poincaré plot at any lag *m *is more of a generalized scenario, where other levels of temporal variation of any dynamic system are hidden.

**Figure 3:**
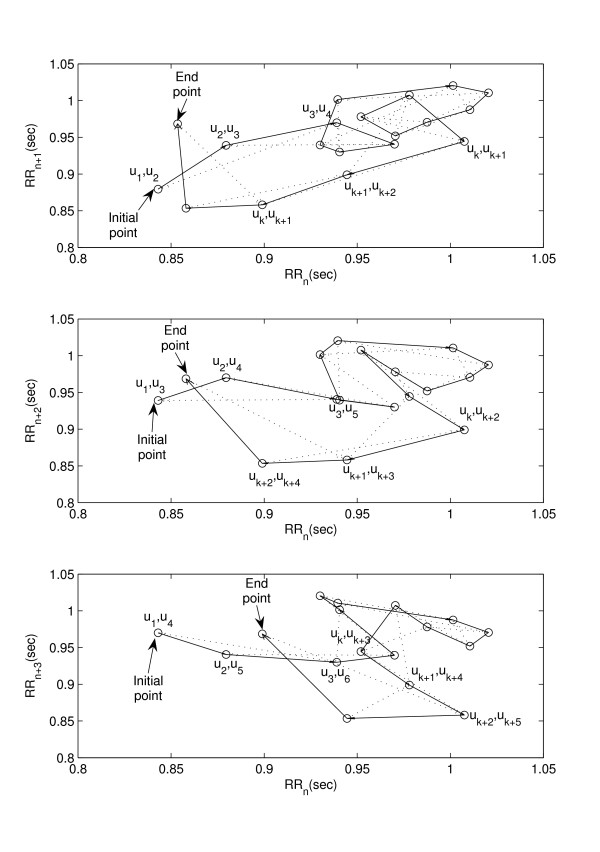
**Forming triangles in calculating *CCM***. Poincaré plot of RR interval time series for three different lags (*m *= {1, 2, 3}) are shown. It is obvious that for RR intervals with different lag plots give more insight of the signal than a single lag. Figure also shows formation of triangles by dotted lines connecting three consecutive points at different lags (*m *= {1, 2, 3}) for 20 RR intervals for calculating *CCM*. Sequence of points (*RR*_*n*_, *RR*_*n*+*m*_) are plotted, where *RR *≡ {*u*_1_, *u*_2_, ......, *u*_*N*_}.

As shown in equation sets 3 and 4, for any *m*, the descriptors *SD*1 and *SD*2 only indicate *m *lag correlation information of the plot. This essentially conveys overall behavior of the system completely neglecting its temporal variation. Figure 3 shows the Poincaré plot of RR interval time series for three different lags. From the figure, it is obvious that for any time series signal different lag plots give more insight of the signal than a single lag plot. Hence, to reflect temporal variation, we developed a descriptor to incorporate multiple lag correlation information, which we call as *Complex Correlation Measure *(CCM). The proposed descriptor is not only related to the standard descriptors, but it also embeds temporal information, which can be used in quantification of the temporal dynamics of the system.

## Methods

The development of a new descriptor, Complex Correlation Measure (CCM) has been presented in this section. Firstly, the theoretical development has been given, followed by the analysis of the new measure with respect to the standard descriptors, SD1 and SD2. Finally, data and methodologies regarding the two case studies have been discussed which is followed by a brief description of statistical analysis used in this study.

### Complex Correlation Measure

In contrast to the *SD*1 and *SD*2, *CCM *evaluates point-to-point variation of the signal rather than gross description of the signal. Moreover, as will be seen later, *CCM *is a function of multiple lag correlation of the signal. The proposed descriptor *CCM *is computed in a windowed manner which embeds the temporal information of the signal. A moving window of three consecutive points from the Poincaré plot are considered and the area of the triangle formed by these three points are computed. This area measures the temporal variation of the points in the window. If three points are aligned on a line then the area is zero, which represents the linear alignment of the points. Moreover, since the individual measure involves three points of the two dimensional plot, it is comprised of at least four different points of the time series for lag *m *= 1 and at most six points in case of lag *m *≥ 3. Hence the measure conveys information about four different lag correlation of the signal. Now, suppose the *i*^*th *^window is comprised of points *a*(*x*1, *y*1), *b*(*x*2, *y*2) and *c*(*x*3, *y*3) then the area of the triangle (*A*) for *i*^*th *^window can be computed using the following determinant:

(5)

where *A *is defined on the real line ℜ and

(6)

If Poincaré plot is composed of *N *points then the temporal variation of the plot, termed as *CCM*, is composed of all overlapping three point windows and can be calculated as:

(7)

where *m *represents lag of Poincaré plot and *C*_*n *_is the normalizing constant which is defined as, *C*_*n *_= π * *SD*1 * *SD*2, represents the area of the fitted ellipse over Poincaré plot. The length of major and minor axis of the ellipse are 2*SD1, 2*SD2, where *SD*1, *SD*2 are the dispersion perpendicular to the line of identity (minor axis) and along the line of identity (major axis) respectively.

Let the RR time series be composed of *N *RR interval values and defined as *RR *≡ *u*_1_, *u*_2_, .., *u*_*N*_. As shown in figure 3, the *lag-1 *Poincaré plot consists of *N *- 1 numbers of 2-D set of points *PP *∈ {ℜ, ℜ} can be represented by: *PP *≡ {(*u*_1_, *u*_2_), (*u*_2_, *u*_3_), .., (*u*_*N*-1_, *u*_*N*_)} and similarly for lag of *m*, *N *- *m *numbers of 2-D points are expressed as:



Hence for *lag-m *Poincaré plot, the 1*st *window will be composed of points {(*u*_1_, *u*_1+*m*_), (*u*_2_, *u*_2+*m*_), (*u*_3_, *u*_3+*m*_)} and from  equation 5, the area *A *can be calculated as:

(8)

Similarly the 2^*nd *^and (*N *- *m *- 2)^*th *^window is composed of points {(*u*_2_, *u*_2+*m*_), (*u*_3_, *u*_3+*m*_), (*u*_4_, *u*_4+*m*_)} and {(*u*_*N*-*m*-2_, *u*_*N*-2_), (*u*_*N*-*m*-1_, *u*_*N*-1_), (*u*_*N*-*m*_, *u*_*N*_)} respectively. Hence the area, *A*, for 2^*nd *^and (*N *- *m *- 2)^*th *^window can be calculated as:

(9)

(10)

Using equations 7, 8, 9 and 10, *CCM*(*m*) is calculated as follows:

(11)

Since RR intervals are discrete signal, the autocorrelation at lag *m *= *j *can be calculated as:

(12)

Using equations 3, 4, 11 and 12, *CCM*(*m*) can now be expressed as a function of autocorrelation at different lags. Hence,



In the above equation *CCM*(*m*) represents the point-to-point variation of the Poincaré plot with lag *m *as a function of autocorrelation of lags 0, *m *- 2, *m *- 1, *m *+ 1 and *m *+ 2. This supports our hypothesis that *CCM *is measured using multiple lag correlation of the signal rather than single lag. For the conventional *lag-1 *Poincaré plot *CCM*(1) can be represented as:

(13)

### Analysis of Complex Correlation Measure(CCM)

In the previous section, we have given the mathematical definition of *CCM *and have clearly shown that *CCM *contains multiple lag correlation information of the signal. In this section, we explore the different properties of *CCM *with synthetic RR interval data. In our study, we used 4000 RR intervals of a synthetic RR interval (*rr02*) time series data from open access Physionet database [16] and the signal was divided into 20 windows with 200 RR intervals in each window.

#### Sensitivity to changes in temporal structure

Literally, the sensitivity is defined as the rate of change of the value due to the change in temporal structure of the signal. The change in temporal structure of the signal in a window is achieved by surrogating the signal (i.e, data points) in that window. In order to validate our assumption we calculated *SD*1, *SD*2 and *CCM *of a RR interval signal by randomly surrogating points of each window at a time.

Now the sensitivity of descriptors Δ*SD*1_*j*_, Δ*SD*2_*j *_and Δ*CCM*_*j *_were calculated using equations 14–16:

(14)

(15)

(16)

where *SD*1_0 _(= 0.36), *SD*2_0 _(= 0.08) and *CCM*_0 _(= 0.16) were the parameters measured for the original data set without surrogation and *j *represents the window number whose data was surrogated. Moreover, *SD*1_*j*_, *SD*2_*j *_and *CCM*_*j *_represents the *SD*1, *SD*2 and *CCM *values respectively after surrogation of *j*^*th *^window. Since we divided the entire signal in 20 windows, it resulted in 20 values of *SD*1, *SD*2 and *CCM*.

#### Sensitivity to various lags of Poincaré plot

To verify the sensitivity of *SD*1, *SD*2 and *CCM *with various lags of Poincaré plot, values of all descriptors were calculated for different time delays or lags (*m *was varied from 1 to 100). At each step, *lag-m *Poincaré plot was constructed for the synthetic RR interval series and then *SD*1, *SD*2 and *CCM *values were calculated for the plot. As *m *was varied from 1 to 100, it resulted in 100 values of *SD*1, *SD*2 and *CCM*.

### Case Studies

In order to validate the proposed measure – *CCM*, two case studies were conducted on RR interval data. The data from MIT-BIH Physionet database are [17] used in the experiments. Medical fraternity has utilized Poincaré plot, using both qualitative and quantitative approaches, for detecting and monitoring arrhythmia. Compared to arrhythmia, fewer attempts are made to utilize Poincaré plot to evaluate CHF. In this study, we have analyzed the performance of *CCM *and compared it with that of *SD*1 and *SD*2 for recognizing both arrhythmia and congestive heart failure using HRV signal.

#### HRV study of Arrhythmia and Normal Sinus Rhythm

In this study, we have used 54 long-term ECG recordings of subjects in normal sinus rhythm (30 men, aged 28.5 to 76, and 24 women, aged 58 to 73) from Physionet Normal Sinus Rhythm database [17].

Furthermore, we have also used NHLBI sponsored Cardiac Arrhythmia Suppression Trial (CAST) RR-Interval Sub-study database for the arrhythmia data set from Physionet. Subjects of CAST database had an acute myocardial infarction (MI) within the preceding 2 years and 6 or more ventricular premature complexes (PVCs) per hour during a pre-treatment (qualifying) long-term ECG (Holter) recording. Those subjects enrolled within 90 days of the index MI were required to have left ventricular ejection fractions less than or equal to 55%, while those enrolled after this 90 day window were required to have an ejection fraction less than or equal to 40%.

The database is divided into three different study groups among which we have used the Encainide (e) group data sets for our study. From that group we have chosen 272 subjects belong to subgroup baseline (no medication). The original long term ECG recordings were digitized at 128 Hz, and the beat annotations were obtained by automated analysis with manual review and correction [17]. *lag-1 *Poincaré plots were constructed for both normal and arrhythmia subjects and the new measure *CCM *was computed along with *SD*1 and *SD*2. The *SD*1 and *SD*2 were calculated to characterize the distribution of the plots whereas *CCM *were used for characterizing the temporal structure of the plots.

#### HRV study of Congestive Heart Failure(CHF) and Normal Sinus Rhythm

For this case study, we have used 29 long-term ECG recordings of subjects (aged 34 to 79) with CHF (NYHA classes I, II and III) from Physionet Congestive Heart Failure database along with 54 ECG recordings of subjects with normal sinus rhythm as discussed earlier [17]. Same ECG acquisition with beat annotations were used as discussed in previous case study. Similar to previous case study, *lag-1 *Poincaré plots were constructed for both normal and CHF subjects and the new descriptor *CCM *was computed as per traditional descriptors.

### Statistical Analysis

In this study we have used ANOVA analysis assuming unknown and different variance for testing the hypothesis regarding mean *i.e.*, the mean of NSR and Arrhythmia groups are equal. It suits our case studies as the sample size is small. The same test has been used to test the hypothesis for NSR and CHF group.

## Results

### Sensitivity to changes in temporal structure

**Figure 4:**
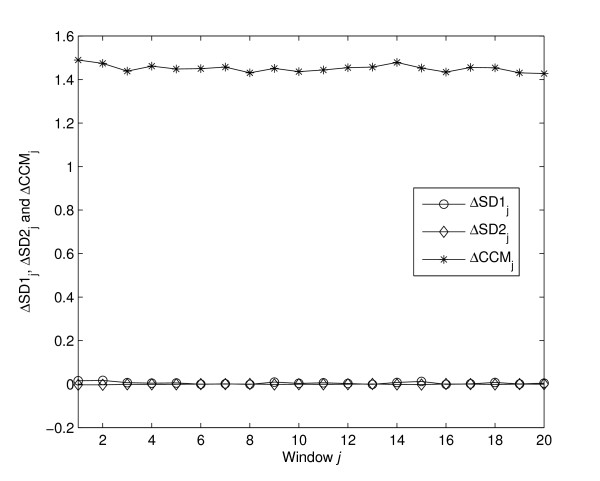
**Sensitivity of descriptors with changed temporal structure**. Sensitivity of all descriptors with change in temporal structure is shown. Δ*SD*1, Δ*SD*2 and Δ*CCM *are calculated using equations 14–16. Value of Δ*CCM *is much higher than Δ*SD*1 and Δ*SD*2 which indicates that *CCM *is much more sensitive than *SD*1 and *SD*2 to the changes in underlying temporal structure of the data.

From the mathematical definition of *CCM*, we anticipated that *CCM *would be more sensitive to changes in temporal structure within the signal than the standard descriptors. In this study, the sensitivity is defined as the rate of change of the value due to the change in temporal structure of the signal. As shown in figure 4, value of Δ*CCM *is much higher than Δ*SD*1 and Δ*SD*2 which indicates that *CCM *is much more sensitive than *SD*1 and SD2 to changes in underlying temporal structure of the data. This supports the mathematical definition of *CCM *as a sensitive measure of temporal variation of the signal.

### Sensitivity to various lags of Poincaré plot

**Figure 5:**
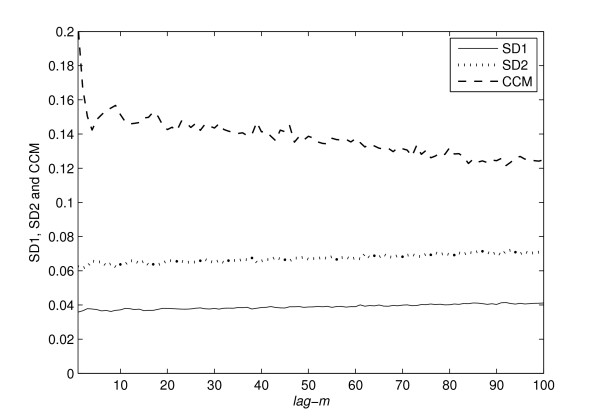
**Relationship of descriptors with different lag**. Relationship of descriptors with different time delays or lags (*m *= 1 to 100) are shown. Values of *SD*1, *SD*2 and *CCM *were calculated for different *lag-m *of Poincaré plot with same number of data points (*N *= 4000). It is found that over different lags, *CCM *shows more variability than *SD*1 and *SD*2.

In figure 5, the relationship of *CCM*, *SD*1 and *SD*2 with different time delays or lags (*m *was varied from 1 to 100) are shown. Usually unit lag is used to create the Poincaré plot which confirms the maximum linear correlation among data points. Moreover, this lag selection may have obscured the low level nonlinearities of the signal and as a result *CCM *may be unable to show better performance over standard poincaré descriptors. In contrast, at higher lags, the standard descriptors are unable to capture the system dynamics. It is also established in the literature that studying behavior of descriptors as a function of lags is more informative [12]. In our study, we have found that over different lags, *CCM *shows more variability than *SD*1 and *SD*2. Among the three descriptors the change in values for *CCM *was higher than both *SD*1 and *SD*2 which again supports our claim of sensitivity of *CCM *with signal dynamics. Hence, we conclude that the change in underlying temporal structure due to lag of the Poincaré plot has better impact on *CCM *than the traditional descriptors.

### HRV studies of Arrhythmia and Normal Sinus Rhythm

**Figure 6:**
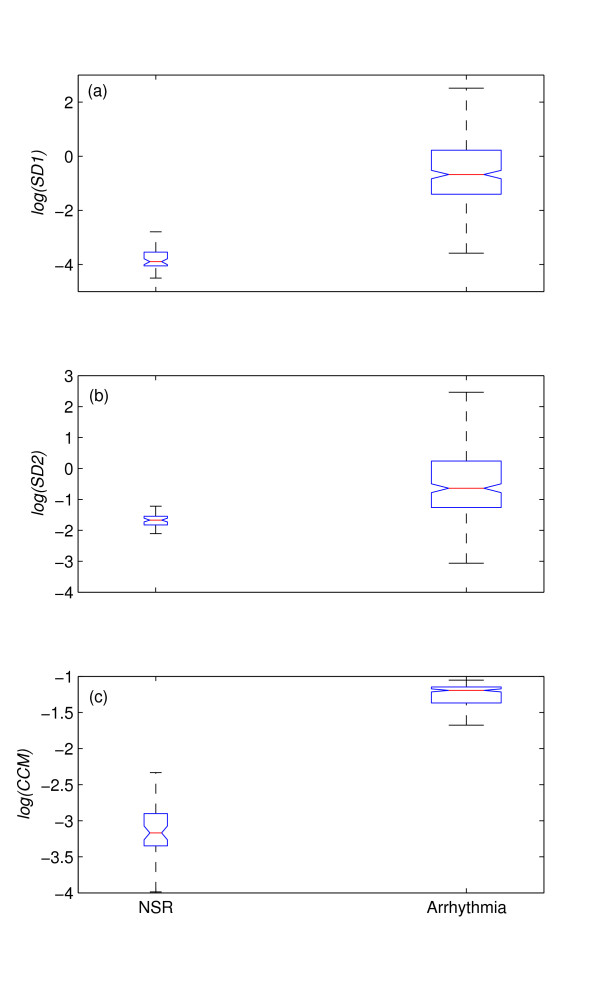
**Distribution of descriptor values for NSR and Arrhythmia subjects**. The distribution of descriptors are shown using Box-whiskers (BW) plot (without outliers) of (a) *log*(*SD*1), (b) *log*(*SD*2) and (c) *log*(*CCM*) for Normal Sinus Rhythm (NSR, n = 54) and Arrhythmia (n = 272) subjects. From panel *a*, it is obvious that boxes (interquartile range) of normal and arrhythmia subjects are non-overlapping. But the whiskers (upper quartile) of normal subjects completely overlaps with the whiskers (lower quartile) of the arrhythmia subjects. From panel *b*, it is apparent that the BW of normal subjects completely overlapped with the whiskers (lower quartile) of the arrhythmia subjects. But the box of arrhythmia subjects is still non-overlapping with the whiskers (upper quartile) of the normal subjects. From panel *c*, it is obvious that both of them are non-overlapping and distinct.

Figure 6 shows the box-whiskers (BW) plot of all descriptors for normal and arrhythmia subjects. For plotting purpose, log value of all descriptors are used for BW plot. Figure 6a, represents BW plot for *log*(*SD*1) and it is obvious that boxes (interquartile range) of normal and arrhythmia subjects are non-overlapping. But the whiskers (upper quartile) of normal subjects completely overlaps with the whiskers (lower quartile) of the arrhythmia subjects. In figure 6b, the BW plot of *log*(*SD*2) is shown and it is apparent that the BW of normal subjects completely overlapped with the whiskers (lower quartile) of the arrhythmia subjects. But the box of arrhythmia subjects is still non-overlapping with the whiskers (upper quartile) of the normal subjects. In figure 6c, the BW plot of *log*(*CCM*) is shown and it is obvious that both of them are non-overlapping and distinct.

The *p *value obtained from ANOVA analysis between two groups for *SD*1, *SD*2 and *CCM *are given in table 1. Using ANOVA, for *CCM*, *p *= 6.28 × 10^-18 ^is obtained whereas for *SD*1 and *SD*2, it is 7.6 × 10^-3 ^and 8.5 × 10^-3 ^respectively. In case of *p *< 0.001 to be considered as significant, only *CCM *would show the significant difference between two groups which indicates that *CCM *is a better descriptor of HRV signal than *SD*1 and *SD*2 when comparing arrhythmia with normal sinus rhythm subjects.

**Table 1:** Mean ± Standard deviation of all descriptors with *p *values for NSR and Arrhythmia subjects

	SD1	SD2	*CCM*
NSR	0.03 ± 0.02	0.19 ± 0.04	0.05 ± 0.03
Arrhythmia	1.92 ± 5.18	2.30 ± 5.86	0.26 ± 0.08

*p *value (ANOVA)	7.60E-3	8.50E-3	6.28E-18

*Mean ± Standard deviation *of *SD*1, *SD*2 and *CCM *for normal sinus rhythm (NSR) and arrhythmia subjects. *p *value from ANOVA analysis is given in the last row. Using ANOVA, for *CCM*, *p *= 6.28 × 10^-18 ^is obtained whereas for *SD*1 and *SD*2, it is 7.6 × 10^-3 ^and 8.5 × 10^-3 ^respectively. In case of *p *< 0.001 to be considered as significant, only *CCM *would show the significant difference between two groups which indicates that *CCM *is a better descriptor of HRV signal than *SD*1 and *SD*2 when comparing arrhythmia with normal sinus rhythm subjects.

### HRV studies of Congestive Heart Failure(CHF) and Normal Sinus Rhythm

**Figure 7:**
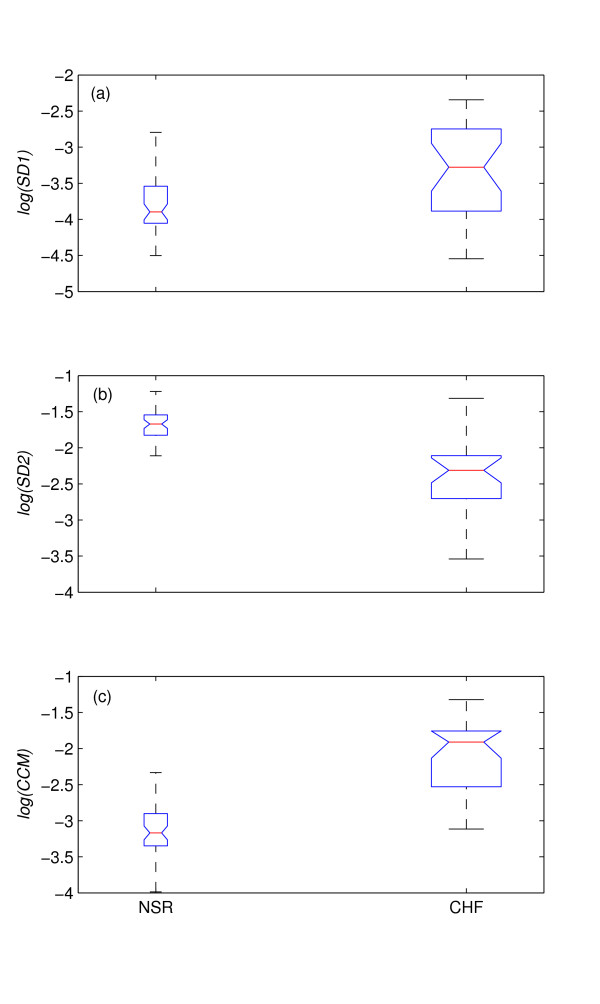
**Distribution of descriptor values for NSR and CHF subjects**. The distribution of descriptors are shown using Box-whiskers (BW) plot (without outliers) of (a) *log*(*SD*1), (b) *log*(*SD*2) and (c) *log*(*CCM*) for Normal Sinus Rhythm (NSR, n = 54) and Congestive Heart Failure (CHF, n = 29) subjects. From panel *a*, it is apparent that boxes (interquartile range) of normal and CHF subjects are overlapping. The BW of normal subjects is completely overlapped with the box and whisker (lower quartile) of the CHF subjects. In panel *b*, boxes are apparently non-overlapped. But the BW plot of normal subjects mostly overlaps with the whisker (upper quartile) of the CHF subjects. In panel *c*, the BW plot of *log*(*CCM*) is shown to be non-overlapping and only the upper quartile (box) and whisker of normal subjects are overlapped with the whisker (lower quartile) of the CHF subjects.

The box-whiskers plot of all descriptors for normal and CHF subjects are shown in Figure 7. Figure 7a, represents BW plot for *log*(*SD*1) and it is apparent that boxes (interquartile range) of normal and CHF subjects are overlapping. The BW of normal subjects is completely overlapped with the box and whisker (lower quartile) of the CHF subjects. In figure 7b, the box-whiskers plot of *log*(*SD*2) is shown and boxes are apparently non-overlapped. But the BW plot of normal subjects mostly overlaps with the whisker (upper quartile) of the CHF subjects. In figure 7c, the BW plot of *log*(*CCM*) is shown to be non-overlapping and only the upper quartile (box) and whisker of normal subjects are overlapped with the whisker (lower quartile) of the CHF subjects.

The values of the mean and the standard deviation for both types of subjects are shown in table 2. Last row represents the *p *value obtained from ANOVA analysis between the two groups for *SD*1, *SD*2 and *CCM*. Though *SD*2 and *CCM *show similar difference between the mean of two subject groups, the *standard deviation *of *CCM *is lower which concentrates with the distribution of *CCM *values around mean comparing with that of *SD*2. The *p *value, obtained from ANOVA analysis for *CCM *(*p *= 9.07 × 10^-14^) shows more significant than *SD*1 and *SD*2.

**Table 2:** Mean ± Standard deviation of all descriptors with *p *values for NSR and CHF subjects.

	*SD*1	*SD*2	*CCM*
NSR	0.03 ± 0.02	0.19 ± 0.04	0.05 ± 0.03
CHF	0.04 ± 0.02	0.11 ± 0.06	0.14 ± 0.06

*p *value (ANOVA)	5.65E-4	5.04E-12	9.07E-14

*Mean ± Standard deviation *of *SD*1, *SD*2 and *CCM *for normal sinus rhythm (NSR) and congestive heart failure (CHF) subjects. *p *value from ANOVA is given in the last row. Though *SD*2 and *CCM *shows similar difference among the mean of two subject groups, the *standard deviation *of *CCM *is lower which concentrates the distribution of *CCM *values around mean comparing with that of *SD*2. The *p *value obtained using ANOVA analysis for *CCM *(*p *= 9.07 × 10^-14^) found more significant than *SD*1 and *SD*2.

## Discussion

The main motivation for using Poincaré plot is to visualize the variability of any time series signal. In addition to this qualitative approach, we propose a novel quantitative measure, *CCM*, to extract underlying temporal dynamics in a Poincaré plot. Surrogate analysis showed that the standard quantitative descriptors *SD*1 and *SD*2 were not as significantly altered as did *CCM*, this is shown in figure 4. Both *SD*1 and *SD*2 are second order statistical measures [2], which are used to quantify the dispersion of the signal perpendicular and along the line of identity respectively. Moreover, *SD*1 and *SD*2 are functions of *lag *- *m *correlation of the signal for any *m *lag Poincaré plot. In contrast, *CCM *is a function of multiple lag (*m *- 2, *m *- 1, *m*, *m *+ 1, *m *+ 2) correlations and hence, this measure was found to be sensitive to changes in temporal structure of the signal as shown in figure 4.

From the theoretical definition of *CCM *it is obvious that the correlation information measured in *SD*1 and *SD*2 is already present in *CCM*. But this does not mean that, *CCM *is a derived measure from existing descriptors *SD*1 and *SD*2. Rather, *CCM *can be considered as an additional measure incorporating information obtained in *SD*1 and *SD*2 (as the lag *m *correlation is also included in *CCM *measure). In a Poincaré plot, it is expected that lag response is stronger at lower values of *m *and it attenuates with increasing values of *m*. This is due to the dependence of Poincaré descriptors on autocorrelation functions. The autocorrelation function monotonically decreases with increasing lags and in case of RR interval time series, usually the current beat influences only about six to eight successive beats [12]. In our study, we also found that all measured descriptors *SD*1, *SD*2 and *CCM *changed rapidly at lower lags and the values are stabilized with higher lag values (figure 5). Since, *CCM *is also a function of signals autocorrelations, it shows a similar lag response to that shown by *SD*1 and *SD*2. Therefore, *CCM *may be used to study the lag response behavior of any pathological condition in comparison with normal subjects, or controls.

HRV measure is considered to be a better marker for increased risk of arrhythmic events than any other noninvasive measure [18,19]. An earlier study has shown that Poincaré plots exposed completely different 2*D *patterns in the case of arrhythmia subjects [20]. These abnormal medical conditions have complex patterns due to reduced autocorrelation of the RR intervals. Consequently due to the changes in autocorrelation, we have found that the variability measure using Poincaré (*SD*1, *SD*2) was higher than normal subjects (shown in table 1). Moreover, the fluctuations of these variability measures were also very high in the case of arrhythmias. This may be due to different types of arrhythmia along with subjective variation of HRV. In arrhythmia subjects, *CCM *was found to be higher compared to NSR subjects, but the deviation due to subjective variation is much smaller than *SD*1 and *SD*2. As a result, *CCM *linearly separates these two groups of subjects which means that the effect of different types of arrhythmia and subjective variation are reduced while using *CCM *than other variability measures. Therefore, we may conclude that *CCM *is a better marker for recognizing arrhythmia than the traditional variability measures of Poincaré plot.

In another case study, we have shown as to how Poincaré plot can be used to characterize CHF subjects from normal subjects using RR interval time series. Compared to *SD*2, *SD*1 and *CCM *values were found to be higher in CHF subjects. This findings might indicate that the short term variation in HRV is higher in CHF subjects, however, the long term variation is reduced. Since *CCM *captures the signal dynamics at short level (i.e, 3 points of the plot), it appears to be affected by short term variation of the signal in CHF subjects. In the case of recognition of CHF subjects, although *SD*2 showed good result *CCM *was found to be more significant as shown in table 2.

Above discussion indicates that *CCM *is an additional descriptor of Poincaré plot with *SD*1 and *SD*2. This also implies that *CCM *is a more consistent descriptor compared to *SD*1 and *SD*2. Considering the presented case studies, it is clear that neither *SD*1 nor *SD*2 alone can independently distinguish between normal and pathology. However, in the same scenario, *CCM *has the ability to perform the classification task independently. This justifies the usefulness of the proposed descriptors as a feature in a pattern recognition scenario. Our primary motivation for detecting pathology with a novel descriptor like *CCM *rather than by observing visual pattern is achieved as shown by the case studies described. In this study, we have not looked at the physiological interpretation of *CCM *which remain to be studied in future. However, a few remarks on this would be appropriate. The Poincaré plot reflects the autocorrelation structure through the visual pattern of the plot. The standard descriptors *SD*1 and *SD*2 summarizes these correlation structure of RR interval data as shown by Brennan *et. al. *[2]. *CCM *is based on the autocorrelation at different lags of the time series hence giving an in-depth measurement of the correlation structure of the plot. Therefore, the value of *CCM *decreases with increased autocorrelation of the plot. In arrhythmia, the pattern of the Poincaré plots becomes more complex [20] and thus reducing the correlation of the signal (*RR*_*i*_, *RR*_*i*+1_). In case of healthy subjects the value of *CCM *is lower than that of arrhythmic subjects. In future, it might be worth looking at the performance of *CCM *for other pathologies.

## Conclusion

The proposed Complex Correlation Measure is based on the limitation of standard descriptors *SD*1 and *SD*2. The analysis carried out confirms the hypothesis that *CCM *measures the temporal variation of the Poincaré plot. In contrast to the standard descriptors, *CCM *evaluates point-to-point variation of the signal rather than gross variability of the signal. We have shown that *CCM *is more sensitive to changes in temporal variation of the signal. We have further demonstrated that *CCM *varies with different lags of Poincaré plot. Besides the mathematical definition of *CCM *and analyzing properties of the measure, we have also evaluated the performance of *CCM *using real world case studies. *CCM *was found to be effective in the assessment of both arrhythmia and CHF against normal sinus rhythm. In future, *CCM *may be used as an efficient feature for pathology detection.

## Competing interests

The authors declare that they have no competing interests.

## Authors' contributions

CKK conceived, derived and implemented the new descriptor, collected the data and wrote the manuscript with supervision of AHK and MP. AHK, JG and MP contributed to the development of the new descriptor and participated in the discussion and interpretation of the results. In addition, AHK contributed to revision of the manuscript. All authors read and approved the final manuscript.
